# Awards, keynotes and gender equity in coastal geoscience and engineering: A 50-year perspective

**DOI:** 10.1017/cft.2026.10033

**Published:** 2026-05-13

**Authors:** Kat Wilson, Sarah Grace Lott, Katherine Anarde

**Affiliations:** 1 Department of Earth and Environmental Sciences, Boston College, USA; 2 Department of Civil, Construction, and Environmental Engineering, North Carolina State University at Raleigh, USA

**Keywords:** awards and keynotes, coastal geoscience and engineering, equity and inclusion, gender equity, workforce diversity

## Abstract

Awards and invited keynotes are critical markers of scholarly achievement that shape visibility, career advancement and retention in academia. Yet extensive evidence across science, technology, engineering, mathematics and medicine shows that women remain underrepresented among recipients of these honors. To evaluate equity within the coastal geosciences and engineering (CGE) community, we compiled the gender distribution of 1,138 awards and invited keynotes granted by professional societies and conferences relevant to CGE over the past 50 years. We additionally reviewed publicly available nomination and selection procedures to assess transparency and inclusivity in award processes. Overall, 65% of honors were presented to men, and 35% were awarded to women and gender-diverse recipients. While men received more than 90% of awards before 2000, female representation among honorees increased to an average of 42% since 2020, outpacing the growth of women in the global scientific workforce and in tenure-track positions in the physical sciences and engineering in the United States. Disparities persist, however, across organizations and career stages: late-career awards and invited keynotes remain disproportionately male-dominated. Most organizations publicly share eligibility criteria, but few provide information on committee composition, evaluation rubrics or conflict-of-interest policies. Our findings show substantial progress toward gender equity in CGE recognition, yet highlight continued gaps in senior-level honors and the transparency of selection procedures. We provide community-focused recommendations such as clearer nomination policies, actions to reduce implicit bias, improved record-keeping and expanded mid-career awards to support equitable recognition across career stages and to ensure that honors reflect the evolving diversity of the CGE workforce.

## Graphical abstract

**Figure:**
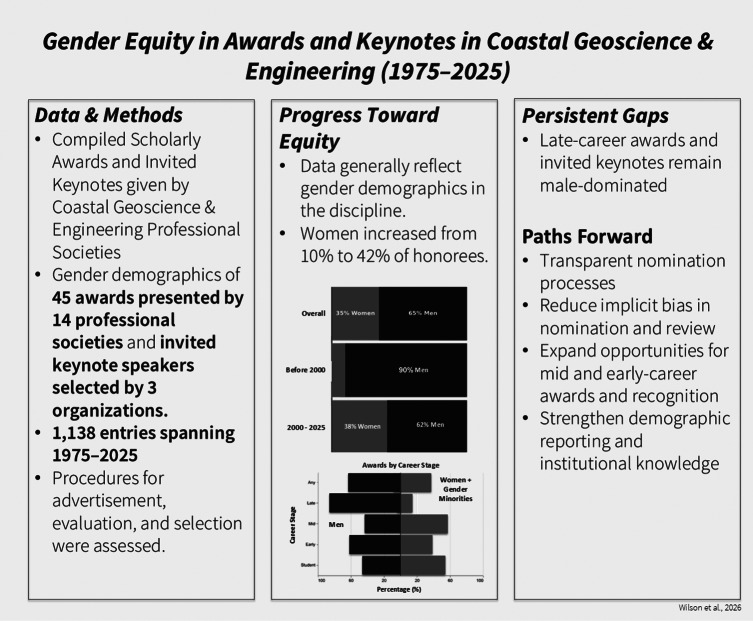

## Impact Statements

This project assembled a database of more than 1,100 entries documenting the gender demographics of honorees within coastal geoscience and engineering (CGE) over the past 50 years. This comprehensive dataset enables evaluation of temporal and organizational trends in gender equity across the field. Our analysis highlights substantial growth in the representation of women and gender minorities (female+) within the CGE workforce. The data show that CGE remains male-dominated; men received 65% of all honors. However, recognition of female+ honorees has increased markedly. Since 2000, their receipt of awards and keynotes has grown at a pace that exceeds their representation in the field, pointing to a possible “catch-up” effect after earlier decades when men received more than 90% of honors. Building on these findings, we offer recommendations for CGE organizations and conferences to strengthen transparency, reduce implicit bias and broaden pathways for recognition in awards and invited keynotes. All compiled data are provided alongside the manuscript. We hope this resource will assist organizations in assessing their internal demographics, awards and selection processes and serve as a foundation for continued progress in equity within CGE.

## Introduction

Scholarly awards and invited keynotes are important for recognizing scientific achievement and advancing careers, particularly in academic research. Evidence across science, technology, engineering, mathematics (STEM) and medicine suggests that women are historically underrepresented as recipients of these honors (Lincoln et al., 2011; Ma et al., 2019; Holmes et al., 2020; Gehmlich and Krause, 2024; RAISE project, 2025). Further, women and gender minorities are less likely to serve as lead authors on manuscripts, be invited to give oral presentations at conferences, conduct peer review and serve in prestigious roles on editorial boards and as conference organizers (Jones et al., 2014; Lerback and Hanson, 2017; Vila-Concejo et al., 2018; Ford et al., 2019; Pico et al., 2020). Meanwhile, women are disproportionately awarded for community service and teaching (Lincoln et al., 2012). In this article, we compile and examine the gender distribution of honorees recognized through named scholarly awards and as invited keynote speakers by scientific societies and conferences relevant to the coastal geosciences and engineering (CGE) community. The global CGE community comprises professionals working across academia, industry and government on coastal processes and engineering topics within core disciplines, including geology, geophysics, geomorphology, physical oceanography, marine science, civil and environmental engineering, planning and management (Vila-Concejo et al., 2018).

Gender demographics and composition of the CGE community are essential for establishing the baseline from which to assess rates of female awardee representation. However, determining the precise gender composition and regional variability is challenging due to the diversity of core disciplines, each of which has distinct reporting standards and representative statistics (e.g., Ranganathan et al., 2021) and limited historical data. Vila-Concejo et al. (2018) reported that across six surveyed CGE organizations (Table 1), female membership ranged from 15 to 45% at the time of the survey. These numbers parallel recent surveys, which report that women are 28–30% of the global STEM research workforce (Huyer, 2015) and 37% of the science and engineering workforce in the United States (National Science Foundation, 2018). Within the largest geoscience professional societies in the United States and Europe, women comprise 28% of the American Geophysical Union (AGU) and 39% of the European Geophysical Union (EGU) memberships (Lerback and Hanson, 2017; Stadmark et al., 2023; Table 1). Further data in the United States show that female participation in engineering fields is ~10–20% less than in the physical and life sciences (Table 1; American Society of Civil Engineers, 2020; National Science Foundation, 2021a), suggesting that within the CGE community, engineering subgroups are more likely to be male-dominated than the other core disciplines (e.g., geosciences and ocean sciences).

**Table 1.** Coastal Geoscience and Engineering (CGE) societies and organizations surveyed in this study and data summary Table 1. long description.

Organization or conference	Acronym	Country/region	Section/division	Female attendance or membership (reference year)	Form of recognition	Years included in analysis	Total entries in dataset
American Geophysical Union	AGU	United States	Ocean Sciences; Earth and Planetary Surface Processes	28%^⇞^ (2015)	Awards	1982–2024	148
American Shore and Beach Preservation Association	ASBPA	United States	–	–	Awards	1988–2024	66
American Society of Civil Engineers	ASCE	United States	–	17%^▲^ (2020)	Awards	2000–2025	204
Australasian Coasts and Ports	AC&P	Australia, New Zealand, and the Pacific Islands	–	–	Awards	2003–2023	42
Australian Coastal Society	ACS	Australia	Coast to Coast, Queensland Coastal Conference, and New South Wales Coastal Conference	45%* (2018)	Keynotes	2007–2025	28
Coastal Estuarine Research Foundation	CERF	United States	–	20%* (2018)	Awards	1997–2023	30
Coastal Sediments and Coastal Dynamics Conferences	CS/CD	International	–		–Awards	2001–2025	15
The Coastal Society	–	United States	–	–	Awards	2002–2024	40
Coasts, Oceans, Ports and Rivers Institute	COPRI	United States	–	15%*	Awards	1978–2025	111
Community Surface Dynamics Modeling System	CSDMS	United States	–		–Awards	2010–2025	27
Estuarine and Coastal Sciences Association	ESCA	United Kingdom	–	38%* (2018)	Awards	2012–2019	5
European Geophysical Union	EGU	Germany / Europe	Ocean Sciences (OS), Geomorphology (GM), Stratigraphy, Sedimentology, and Paleontology (SSP)	39%^+^ (2022)	Awards	1991–2025	190
International Conference on Coastal Engineering	ICCE	International	–	–	Keynotes	1998–2024	48
National Institute of Maritime, Port, and Aviation Technology Port and Airport Research Institute	NIA	Japan	–	–	Awards	2016–2024	17
New Zealand Coastal Society	NZCS	New Zealand	–	40%* (2018)	Awards	2012–2024	31
The World Association for Waterborne Transport Infrastructure	PIANC	International	–	–	Awards	1985–2024	74
Rivers, Coasts and Estuarine Morphodynamics Conference	RCEM	International	–		–Keynotes	1999–2025	78

*Note:* Female attendance and membership statistics, where available, come from Vila-Concejo et al. (Vila-Concejo et al., 2018) (*), Lerback and Hanson (Lerback and Hanson, 2017) (⇞), American Society of Civil Engineers (American Society of Civil Engineers, 2020) (▲) and Stadmark et al. (Stadmark et al., 2023) (+).

**Table 2.** Summary of publicly available information on nomination and selection processes for organizational awards Table 2. long description.

Organization	Award	Style of award	Advertisement	Nomination				Evaluation					Source
			Open nomination with publicized regular due date	Eligibility requirements for the nominee	Eligibility requirements and role of the nominator	Established policies for extension of eligibility	List of supporting materials needed for the package	Stated evaluation structure (committee, panel, etc.)	If by committee, the size and composition of the committee is public	Evaluation criteria or rubric is shared	Advertised list of previous awardees	Conflict of interest policy	
ASBPA	Rising Star	Early Career	X	X			X				X		ASBPA (American Shore and Beach Preservation Association, 2024)
ASBPA	Morrough P. O’Brien	Significant Achievement	X	X			X				X		ASBPA (American Shore and Beach Preservation Association, 2024)
ASBPA	President’s Award	Significant Achievement	X	X			X				X		ASBPA (American Shore and Beach Preservation Association, 2024)
ASBP	Robert G. Dean Coastal Academic Award	Significant Achievement	X	X			X				X		ASBPA (American Shore and Beach Preservation Association, 2024)
ASBPA	Student Poster	Self-Nominated Paper or Presentation	X	X			X				X		ASBPA (American Shore and Beach Preservation Association, 2024)
ASCE	Hans Albert Einstein Award	Nominated Award	X	X	X		X	X	X		X		ASCE (American Society of Civil Engineers, 2025a, 2026)
ASCE	Edmund Friedman Young Engineer Award for Professional Achievement	Early Career	X	X	X		X	X	X		X		ASCE (American Society of Civil Engineers, 2025b, 2026)
ASCE	Edmund Friedman Professional Recognition Award	Significant Achievement	X	X	X		X	X	X		X		ASCE (American Society of Civil Engineers, 2025c, 2026))
ASCE	Daniel W. Mead Prize for Young Members	Early Career	X	X	X		X	X	X		X		ASCE (American Society of Civil Engineers, 2025d, 2026)
ASCE	Daniel W. Mead Prize for Students	Student	X	X	X		X	X	X		X		ASCE (American Society of Civil Engineers, 2025e, 2026)
AGU	Ocean Sciences Award	Significant Achievement	X	X	X	N/A	X	X	X		X	X	AGU (American Geophysical Union, 2025a, 2025b)
AGU	Harald Sverdrup Lecture	Nominated Lecture	X	X	X		X	X	X		X	X	AGU (American Geophysical Union, 2025a, 2025c)
AGU	G.K. Gilbert Award In Surface Processes	Significant Achievement	X	X	X		X	X	X		X	X	AGU (American Geophysical Union, 2025a, 2025d)
AGU	Ocean Sciences Voyager Award	Mid-career	X	X	X	X	X	X	X		X	X	AGU (American Geophysical Union, 2025a, 2025e)
AGU	Rachel Carson Lecture*	Limited to Female Awardees; Nominated Lecture	X	X	X		X	X	X		X	X	AGU (American Geophysical Union, 2025a, 2025f)
AGU	William S. and Carelyn Y. Reeburgh Lecture	Nominated Lecture	X	X	X		X	X	X		X	X	AGU (American Geophysical Union, 2025a, 2025g)
AGU	Ocean Sciences Early Career Award	Early Career	X	X	X	X	X	X	X		X	X	AGU (American Geophysical Union, 2025a, 2025h)
AGU	Luna B. Leopold Early Career Award	Early Career	X	X	X	X	X	X	X		X	X	AGU (American Geophysical Union, 2025a, 2025i)
Australasian Coasts and Ports	DN Foster	Student; Self-Nominated Paper or Presentation	X	X	N/A		X				X		Australasian Coasts and Ports Conference Series (2025)
Australasian Coasts and Ports	WICGE*	Limited to Female Awardees; Self-Nominated Paper or Presentation	X	X	N/A		X			X			Australasian Coasts and Ports Conference Series (2025)
CERF	Cronin Award	Early Career		X	X		X	X			X	X	CERF (Coastal and Estuarine Research Foundation, 2025)
CERF	Odum Award	Significant Achievement		X	X		X	X			X	X	CERF (Coastal and Estuarine Research Foundation, 2025)
Coastal Sediments / Coastal Dynamics	Coastal Award	Significant Achievement									X		Coastal Sediments (2026)
Coastal Society	Robert W. Knecht Award for Professional Promise	Early Career									X		The Coastal Society (n.d.)
Coastal Society	Student Poster Award	Self-Nominated Paper or Presentation									X		The Coastal Society (n.d.)
COPRI	John G. Moffatt–Frank E. Nichol Harbor and Coastal Engineering Award	Significant Achievement	X	X			X	X	X		X		ASCE (American Society of Civil Engineers, 2025f, 2026)
COPRI	Orville T. Magoon Sustainable Coasts Award	Nominated Award	X	X			X	X			X		ASCE (American Society of Civil Engineers, 2025g, 2026)
COPRI	Kenneth M. Childs, Jr. Practitioner’s Award	Significant Achievement	X	X			X	X			X		ASCE (American Society of Civil Engineers, 2025h)
COPRI	International Coastal Engineering Award	Significant Achievement	X	X			X	X			X		ASCE (American Society of Civil Engineers, 2025i, 2026)
CSDMS	Student Awards	Self-Nominated Paper or Presentation	X				X	X		X	X		CSMDS (Community Surface Dynamics Modeling System, 2025a)
CSDMS	Late career	Significant Achievement					X				X		CSMDS (Community Surface Dynamics Modeling System, 2025b)
ECSA	Lifetime achievement	Significant Achievement	X	X	X		X				X	X	ECSA (Estuarine & Coastal Sciences Association, 2025)
EGU	Jean Baptiste Lamarck Medal	Significant Achievement	X	X	X		X				X	X	EGU (European Geophysical Union, 2025a, 2025b, 2025c)
EGU	Ralph Alger Bagnold Medal	Significant Achievement	X	X	X		X				X	X	EGU (European Geophysical Union, 2025a, 2025b, 2025c)
EGU	Fridtjof Nansen Medal	Significant Achievement	X	X	X		X				X	X	EGU (European Geophysical Union, 2025a, 2025b, 2025c)
EGU	Outstanding Student Presentation	Student	X	X	X		X				X	X	
EGU	Arne Richter Award for Outstanding Early Career Scientists	Early Career	X	X	X		X				X	X	EGU (European Geophysical Union, 2025a, 2025b, 2025c, 2025d)
EGU	Outstanding Early Career Scientist	Early Career	X	X	X	X	X	X	X		X	X	EGU (European Geophysical Union, 2025a, 2025b, 2025c, 2025d)
National Institute of Maritime Port and Aviation Technology. Port and Airport Research Institute	Hamaguchi Award	Significant Achievement	X	X			X	X			X		National Institute of Maritime, Port, and Aviation Technology, Port and Airport Research Institute (National Institute of Maritime, Port, and Aviation Technology, Port and Airport Research Institute, 2025)
NZCS	Maori & Pacific Island Research Scholarship; Student Research Scholarship: Masters; Student Research Scholarship: PhD	Scholarships	X	X	X			X	X	X	X		New Zealand Coastal Society (2025)
PIANC	YP award	Self-Nominated Paper or Presentation	X	X	N/A	X	X	X		X	X		PIANC (2025a)
PIANC	International De Paepe-Willems Award	Self-Nominated Paper or Presentation	X	X	N/A		X	X	X	X	X		PIANC (2025b)
PIANC	ANZ Young Authors Award	Self-Nominated Paper or Presentation	X	X	N/A		X				X		PIANC Australia and NZ (2025)

*Note:* An “X” denotes information that was accessible at the time of data collection, gray indicates information was not available and “N/A” signifies that the category was not applicable (e.g., nominator role in cases of self-nomination). *Denotes an award limited to female recipients.

Data on the demographics of women within different career stages among the CGE community are also lacking. However, evidence from broader STEM fields shows that women are more strongly represented at student and early-career stages than at senior levels, and that female participation in the US-based STEM workforce has increased dramatically over the past 50 years (National Science Foundation, 2018). Degree attainment and faculty demographic data, in part, illustrate this trend: in the Earth and atmospheric sciences, women’s share of degrees rose from 10% of bachelor’s and 6% of master’s degrees in 1967 to 38% and 43% in 2018, respectively, with women earning 44% of doctoral degrees in 2018 (National Science Foundation, 2021a, 2021b). In engineering, the proportion of doctoral degrees conferred to women increased from 12% in 1998 to nearly 25% in 2018 (National Science Foundation, 2021a, 2021b). In 1973, women were absent from engineering faculty ranks and represented only 5% of junior and 3% of senior faculty across the physical sciences in the United States (National Science Foundation, 2018).

Recent studies report that among US Earth science departments, women hold 35–46% of assistant, 43% of associate and only 13–20% of full professor positions (Glass, 2015; Ranganathan et al., 2021). Across engineering fields, women comprise 22% of junior and 14% of tenured faculty (National Science Foundation, 2018). These trends are mirrored in the European Union, where women are awarded 36% of doctoral degrees in natural sciences and engineering, but decline in representation at higher career ranks (Schroeder et al., 2013; European Commission, 2025). Yet these overall gains are tempered by lower rates of tenure and promotion (National Academies Press, 2006; European Commission, 2025), persistent attrition and the historical exclusion of women, gender and sexual minorities and people of color. As a result, participation in Earth sciences and engineering remains well below the representation of these demographic groups in the broader population (Holmes et al., 2008; Dutt et al., 2016; Lerback and Hanson, 2017; Bernard and Cooperdock, 2018; Hughes, 2018; Dutt, 2020; Rathburn and Ely 2021).

Recognition through honors may play an important role in faculty retention. Honors contribute directly to tenure and promotion decisions and help build a reputation of scholarly respect. This recognition enhances self-efficacy and increases visibility among colleagues and junior scientists. In turn, such visibility serves as an aspirational signal for those entering the field, shaping their perception of possible career trajectories (Rice et al., 2000; De Welde and Laursen, 2011). As discussed above, women are less likely to be nominated for and receive scholarly honors across STEM fields. A well-documented source of exclusion and bias lies within the nomination and selection processes themselves (Holmes et al., 2020). Factors such as limited transparency, implicit bias, the composition of selection committees and conflicts of interest embedded in the advertisement, nomination materials and review procedures can all strongly influence outcomes (Lincoln et al., 2009, 2012; Lagisz et al., 2023, 2024). Thus, systematic evaluation of how scholarly awards and honors are distributed is essential to ensuring equity in academic recognition and advancement.

We are three early-career, cisgender women in the CGE community, and we are neither eligible nor in positions to nominate others for many of the honors included in this study. As junior scientists, we also have not served on the organizing committees of the conferences or selection committees of any of the awards examined here. Our interest in conducting this work stems from our roles in the Women in Coastal Geoscience and Engineering (WICGE) organization and our commitment to improving transparency and supporting the broader CGE community. This study was motivated by the question: Are women and gender minorities being awarded and recognized proportionally to their participation in CGE organizations? To answer this question, we (1) compile and quantify the gender distribution of scholarly honors given by CGE organizations, (2) evaluate temporal trends over the past 50 years and (3) examine whether gender demographics of awardees vary with the transparency and equity of the nomination and selection procedures. This analysis provides baseline data for the CGE community to assess gender representation among awardees relative to organizational demographics and to review nomination, evaluation and selection procedures that support diverse candidate pools and equitable recognition.

## Methods

We compiled a dataset of the gender demographics of the recipients of scholarly and technical honors in the CGE community within the past 50 years (1978 through August 2025). In our analysis herein, we include only scholarly honors that have been presented for scientific and engineering merit at least five times since 1978. This analysis considers nominated significant achievements and early career awards, invited keynote speakers and named lectures, best student presentation or paper awards and grants for student research and conference attendance (Supplemental Material SM1). Within our dataset, all student awards were for conference presentations, papers or small scholarships. Instances of participation on plenary panels, honorable mentions in student competitions and invited oral presentations within conference sessions were not available and, therefore, were excluded from the dataset. Awards for service to the community or organization were also compiled (Supplemental Material SM1) but were excluded from the analysis presented here. Service awards are known to be distributed differently across genders and career stages and often recognize forms of labor and contributions that are not directly comparable to scholarly achievement (Lincoln et al., 2012). Therefore, including them in the primary analysis would conflate distinct categories of recognition and complicate the interpretation of patterns related specifically to scientific merit.

**Figure 1.**
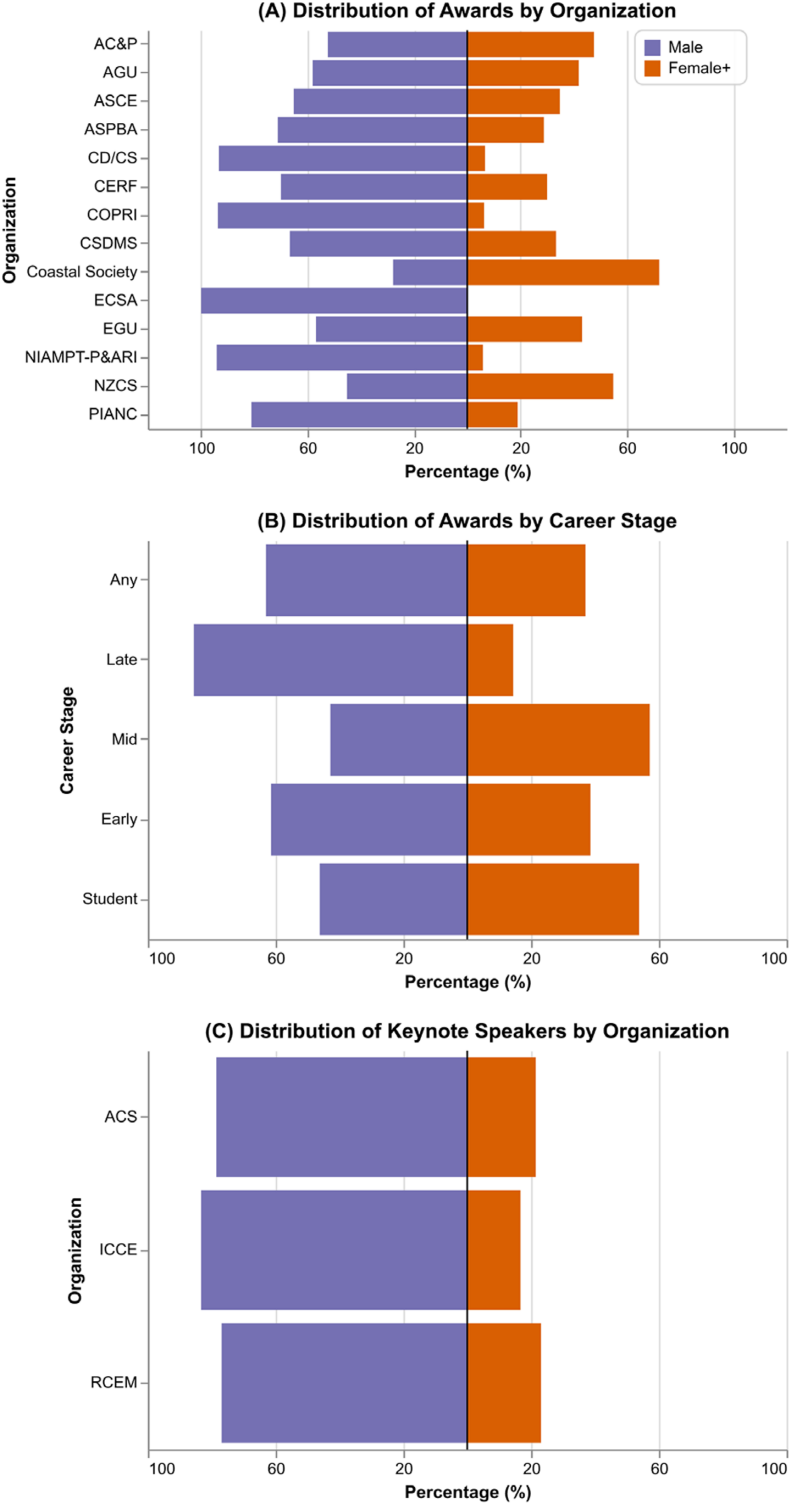
Diverging bar plots of the percentage of the total scholarly honors conferred to men versus women and gender minorities within the CGE community (1978–2025), A) awards presented by organization, B) awards given by career stage, and C) invited keynote speakers by organization. Figure 1. long description.

The societies and conferences included in our analysis (Table 1) span the diversity in core disciplines characteristic of the CGE community, and the awardees include individuals within different sectors of local to federal governments, industry and academia. The dataset includes 984 instances of 45 awards given by 14 organizations and 154 invited keynote speakers from five conferences, which lack established awards. Three regional conferences of the Australian Coastal Society (ACS) were combined into one entry representing the broader organization (Table 1; Figure 1C). The dataset includes two honors (*n* = 30), which can only be received by women. We conducted a review of published literature to assemble available records of female participation across societies and organizations in the dataset (Lerback and Hanson, 2017; Vila-Concejo et al., 2018; American Society of Civil Engineers, 2020; Stadmark et al., 2023; Table 1).

Most data were obtained from organizational websites (Tables 1 and 2; Supplemental Material SM1). For each record, we compiled the name of the awarding society, award name, years conferred and the name of the awardee (Wilson et al., 2026). The society section and career stage associated with the award were recorded when applicable. Institutional affiliation at the time of award or nationality of winners were not available with the award announcements. When records were missing, conference organizers or senior members of the society or organization were contacted directly (Supplemental Material SM1). Notably, the record of keynote speakers contains more data gaps than the award record, primarily due to the absence of available documentation from conference organizers or published programs. For large organizations such as the AGU and EGU, we focused on honors in divisions most relevant to the CGE community (e.g., Ocean Sciences, Earth and Planetary Surface Processes); however, awardees in these sections may not all identify as CGE researchers.

Additionally, we compiled available qualitative data regarding eligibility and nomination processes for awards via organizational websites and by contacting conference organizers (Table 2). We summarize the availability of information provided by each organization or society across three stages of the award process: advertisement, nomination and evaluation/selection. The summarized data reflect the policies and procedures at the time of preparation of this manuscript and, therefore, do not necessarily apply to all years in which an award was conferred. The invited keynote data are excluded from Table 2. Per communication with conference organizers, invited keynotes at CGE conferences are most commonly selected by local organizing committees and lack established guidelines or shared policies across years.

Gender classification of honorees – *male* or *female and gender minorities* (herein, “female+”) – was carefully determined using names, photos and self-identified pronouns on public-facing materials (e.g., professional webpages, university profiles or other self-descriptive text). In cases where these details could not be found, the gender of the individual was inferred using name-based gender associations relying on the common usage in the cultural context of the individual (Blevins and Mullen, 2015; Lagisz et al., 2024). Although we sought to recognize the presence and contributions of gender minorities within the CGE community, honors given to gender-diverse individuals were ultimately grouped with female awardees due to the very small sample size and the associated statistical limitations. We also acknowledge that some individuals may have been inaccurately classified or undercounted within the female+ category if their gender identity is not disclosed or visible in their professional lives (e.g., Clair et al., 2005; Duncombe, 2019; Powell et al., 2020).

Awards are presented by career stage. The dataset includes nine student awards (*n* = 236), which primarily recognize graduate students for conference presentations or best papers, or are small monetary scholarships for research and conference attendance. Student awards may occasionally include undergraduate recipients. Early-career awards in the dataset are conferred to post-doctoral fellows, researchers and professors within 5–10 years of completing a doctoral degree. In limited cases, early-career eligibility policies allow for extension to account for time away from scientific activities due to caregiving, illness or comparable responsibilities (AGU, 2025a; EGU, 2025d). Such provisions are designed to ensure that scientists are not disadvantaged when early-career stages overlap with family or care-related obligations and are a benefit to all genders. Early-career awards represented the largest category in this analysis, with 306 entries spanning 13 distinct awards. The only mid-career award included in the dataset is the “Ocean Voyagers Award” from the AGU Ocean Sciences section (*n* = 7), designated for scientists 10–20 years past their doctorate. “Significant” or “lifetime achievement” awards were the most common category (*n* = 281 across 16 awards). These typically have no formal eligibility criteria and are most often awarded to senior scientists and full professors (“late” or “experienced” career stage). An additional six awards and named lectures (*n* = 152) lacked specified career stages.

The surveyed organizations are largely based in the United States, Europe and Australasia (Table 1); therefore, we acknowledge our analysis is not geographically comprehensive. However, these organizations are open to global researchers and attract international participation. A full assessment of the nationality of awardees is beyond the scope of this study, but future work could explore biases favoring the Global North in international academic awards.

All analyses and calculations were conducted within Microsoft Excel (Microsoft Corporation, 2026). Initial drafts of figures were generated with Python (Lott, 2026) and manually edited in Adobe Illustrator (Adobe, 2026).

## Results

**Figure 2.**
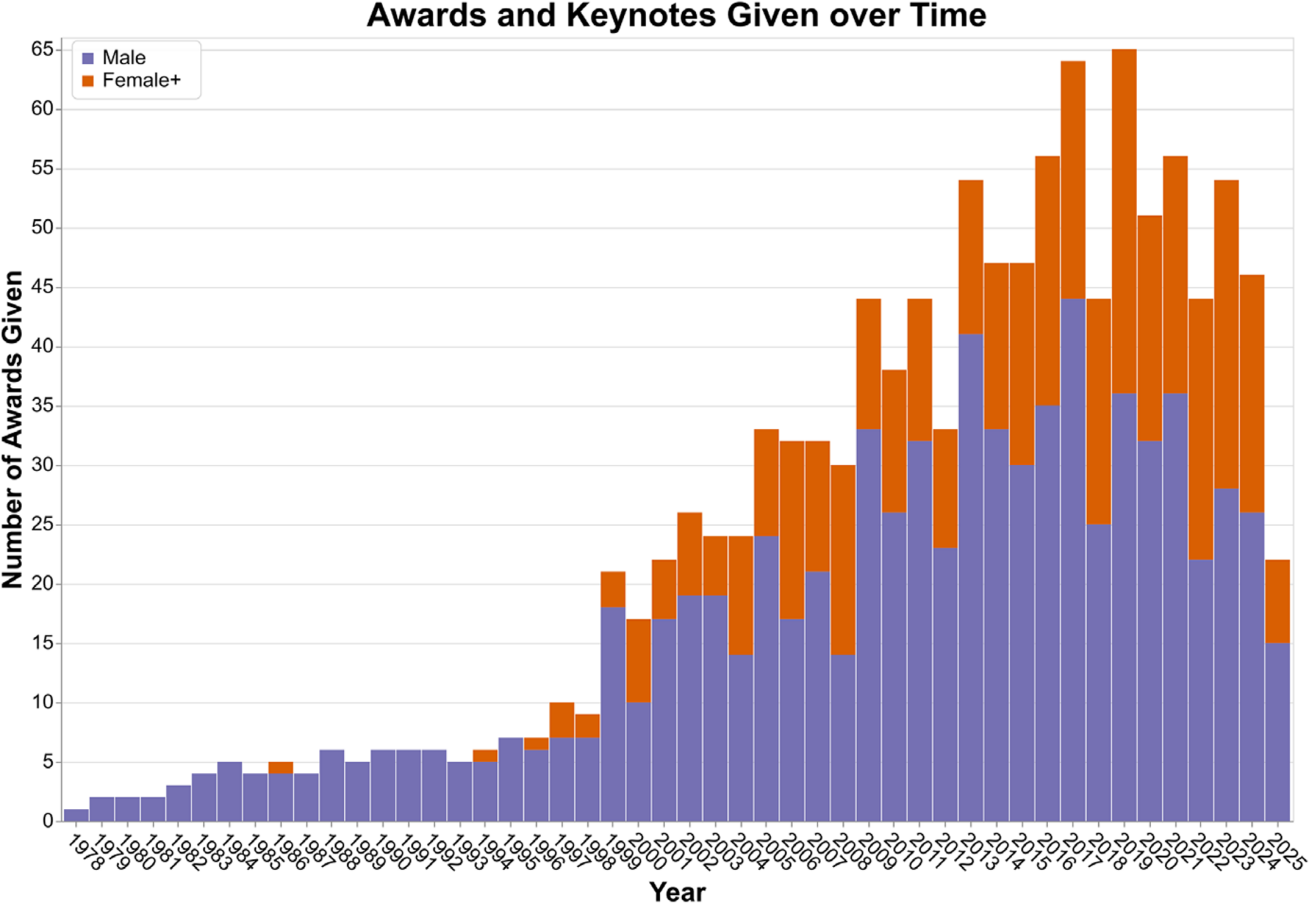
Summed awards and keynotes given by year, combining all organizations and career stages. Honorees are categorized as male or female and gender minorities. Figure 2. long description.

Overall, in the past 50 years, 65.5% of the recorded awards and invited keynotes were presented to male and 34.5% to female+ honorees (Figure 1A). We observe temporal trends, with men receiving an average of 94.4% of scholarly awards between 1978 and 1999, compared to 62% between 2000 and 2025 (Figure 2). The year 2000 also coincides with a step change in total awards given per year, which increased from an average of 5–25 per year (Figure 2). The earliest recorded award received by a woman in the dataset occurred in 1986, followed by a subsequent 9-year gap before the next appearance of a female awardee in 1994 (Figure 2). The percentage of female and gender-diverse CGE awardees has outpaced the growth of women in tenure-track positions in the physical sciences and engineering in the United States (National Science Foundation, 2018; Supplemental Material SM2). Female and gender diverse representation among honorees increased from an average of 0% in the 1970s to 2.5% in the 1980s, 14.3% in the 1990s, 38.4% in the 2000s, 34.9% in the 2010s and 42.1% between 2020 and 2025. The proportion of female+ honorees peaked in 2022, when women and gender minorities received 61.5% of total honors (*n* = 16 of 26 awards and keynotes).

Five organizations have overall recipient gender ratios more than 1 standard deviation above the mean (>77%:23% male: female+ ratio): Coastal Sediments/Dynamics (93%:7%), PIANC (85%:15%), NIAMPT-P&ARI (94%:6%), COPRI (94%:6%) and ECSA (100%:0%). Only one organization has gender ratios more than one standard deviation below the mean (<43% male): The Coastal Society (18%:82%).

When disaggregated by career stage (Figure 1B), male: female+ awardee ratios were as follows: student (46%:54%), early-career (61%:39%), mid-career (43%:57%) and late career (86%:14%). Awards without specified career stages aligned with the overall average male: female+ ratio of 65%:35%. Female+ awardees outnumber male recipients in the student awards (*n* = 236) and mid-career stage (*n* = 7).

Among the 154 invited keynotes, 79.2% were delivered by men and 20.8% by women and gender minorities (Figure 1C). Keynotes are disproportionately male-dominated, and it is likely that missing historical data would further accentuate this disparity. Conference organizers of RCEM and ICCE who were contacted could not recall any female keynote speakers before 2000.

The dataset of awards showed 96% of entries were first-time recipients of awards. The data showed a small number of awardees who appeared twice in the dataset, and only one individual received three awards. Keynotes have a higher rate of repeated invitations (9%), but 91% of the entries represent first-time invitees.

Available information on advertisement, eligibility and selection procedures is reported for each of the 45 distinct awards in the dataset (Table 2, Supplemental Material SM4). The dataset was limited to awards and honors for which past recipients were publicly listed on society webpages, enabling assessment of awardee gender demographics. Most awards (39/45) listed open nomination windows or published deadlines. However, six awards provided no information on nomination timing or procedures, which suggests that nominations may rely on informal networks or communication.

Eligibility and nomination criteria were typically published (40/45), though descriptions of achievement were often vague and relied on subjective language (e.g., “outstanding and sustained contributions”), which may perpetuate implicit bias in lifetime or significant achievement awards. Requirements related to nominator seniority or society membership were specified in roughly half of the cases (22/45).

Information on evaluation and selection processes was less commonly available (Table 2; Supplemental Material SM4). Explicit evaluation criteria or scoring rubrics were provided only for student papers or scholarship awards (7/45). Conflict-of-interest policies were publicly available for three societies (AGU, EGU, and CERF). Selection committee composition was disclosed for half of the cases; named committee members were available only for AGU and EGU (24/45). Policies regarding implicit-bias training for selection committees, as recommended in prior studies (Lincoln et al., 2012; Holmes et al., 2020), were not publicly available for any award.

## Discussion and paths forward

Female+ awardees in CGE organizations (35%, Figure 1A) generally reflect – and in some cases exceed – women’s participation in the CGE community (15–48%, Table 1) and the broader STEM workforce (28–30%, [Huyer, 2015]). The rise in female awardees over time (Figure 2) parallels the steady increase of women in graduate programs, tenure-track faculty roles and the CGE workforce over the past five decades, which has expanded the pool of eligible candidates. Notably, the growth in female+ awardees has outpaced the increase in female+ tenure-track faculty in physical sciences and engineering in the United States (Supplemental Data SM2, SM3), even when student awards are excluded. For example, since 2020, women and gender minorities have accounted for an average of 42% of honorees. This trend suggests that early studies identifying gender disparities, along with policy changes in large organizations over the past two decades, may be contributing to more representative candidate pools and award outcomes. Recent increases may also partially reflect efforts to “catch up” for years of under-recognition. However, female and gender-diverse representation remains uneven across conferences and organizations (Figure 1A; Supplemental Material SM3), highlighting ongoing opportunities for improvement.

Late-career awards follow expected trends based on academic demographics, with fewer senior women recognized across organizations, consistent with historically smaller cohorts. Given their prominence and broad visibility within conferences and scientific societies, male-dominated lifetime achievement awards and invited keynotes may disproportionately shape perceptions of gender imbalance within CGE, even though these awards and the dataset as a whole broadly align with estimated participation demographics. There are two career stages where women are receiving awards at higher rates than their male colleagues: student awards and the single mid-career award. In addition to the growing number of women in these age cohorts, we suggest additional factors that may contribute to this outcome. In our dataset, student awards are often self-nominated (Table 2), which reduces gender bias frequently associated with nomination letters, where studies have shown that women are often described with fewer superlatives and more personal details (Lincoln et al., 2012; Dutt et al., 2016). The mid-career pattern may be a result of the small sample size, as this category includes only one AGU award with fewer than 10 recipients since 2014. Its recency also avoids decades of male-dominated outcomes.

Our data reflect the current state of award nomination and selection processes in 2025 (Table 2), which may already incorporate recommendations for inclusivity proposed over the past two decades (e.g., National Academies Press, 2006; Vila-Concejo et al., 2018; Holmes et al., 2020). Therefore, there is not necessarily a direct or easy correlation to be made between the summary gender demographics and the nomination processes presented here. However, past studies indicate that transparency in policy and process leads to more equitable outcomes and higher female representation among awardees, and those conclusions are supported by our data. For instance, some organizations maintain transparent, publicly accessible nomination and evaluation procedures. AGU and EGU, for example, have consistent nomination cycles and established conflict-of-interest policies. These organizations exceed the CGE average in gender representation and are approaching overall gender parity among award recipients. In contrast, smaller, locally organized and volunteer-led conferences often use less formal, ad hoc nomination processes. These organizations tend to show wider disparities (Figure 1A, Table 2).

Notably, invited keynotes exhibit the widest gender disparity and a higher incidence of repeat honorees (Supplemental Material SM1). Keynotes are typically chosen by organizing committees without formal guidelines, suggesting that professional and social networks, and prior recognition by other professional societies, may influence selection. Prior studies indicate that women are more likely to decline speaking invitations, often due to logistical challenges such as caregiving responsibilities, limited childcare support at conferences and gendered differences in self-promotion and self-efficacy (Pray, 2003; Schroeder et al., 2013). We could not assess the frequency of declined invitations in this study because data on initial invitations versus finalized keynote schedules were not available. In practice, achieving parity may be difficult even when invitations are distributed equitably. Nevertheless, because keynote speakers are highly visible and function as public indicators of whose work is valued and elevated within the field, their selection represents a critical opportunity for intentional improvement. If gender parity is a stated goal, organizers may need to expand recruitment beyond existing networks via canvassing committees and extend invitations more broadly to ensure that highly qualified women are not overlooked.

Smaller, volunteer-led organizations and conferences may face logistical challenges that affect gender equity in awards. Maintaining published databases of past honorees and coordinating consistent nomination and selection processes requires time and monetary resources. For example, while student paper awards are common at CGE conferences, records of past recipients were often unavailable during our data compilation, so several student awards could not be included in our analyses. Despite these challenges, transparency in publicizing award cycles, nomination procedures and evaluation processes likely supports the development of an equitable applicant pool that reflects the broader CGE membership (Holmes et al., 2020; Lagisz et al., 2024).

Similar analyses have recently been conducted in other scientific disciplines, where comparable structural patterns in recognition and awards have been documented. The convergence of findings across fields suggests that these patterns are not discipline-specific but reflect broader systemic gender dynamics and biases (Schroeder et al., 2013; Jones et al., 2014; Lagisz et al., 2023; Lagisz et al., 2024). Previous studies have provided thoughtful and actionable recommendations for scientific societies to close the gender gap in scholarly awards, best paper competitions and prestige roles (Vila-Concejo et al., 2018; Greider et al., 2019; Holmes et al., 2020; Lagisz et al., 2024). Below, we echo these recommendations and note how they can be made relevant to the CGE community. Societies may adopt or implement recommendations at different paces depending on their size, resources and governance structure, particularly in volunteer-led organizations. These recommendations are meant to be aspirational and modular, rather than prescriptive or all-or-nothing: incremental or piece-wise actions can still represent meaningful progress. Importantly, while racial, ethnic and geographic diversity in awardees was not reported in this study, progress toward more inclusive and transparent policies, such as those recommended here, is expected to broadly improve outcomes for underrepresented and marginalized groups in CGE. Finally, we do not advocate for the creation of additional demographic-restricted awards (e.g., women-only awards), as these may inadvertently create parallel tiers of recognition (Lincoln et al., 2012). Instead, efforts should focus on increasing the nomination and selection of diverse individuals for open, unrestricted awards.

Five recommendations for improving gender equity in CGE awards and keynotes

1. *Transparency in the nomination and selection policies.* Nomination and selection policies should be regularly reviewed to ensure transparency, inclusivity and fairness. At a minimum, award materials should clearly specify the nomination deadline, required documents and the evaluation criteria so that potential nominators and nominees at all career stages understand eligibility and expectations. Further, policies for extension of eligibility (e.g., for early-career awards) should be clearly stated, and the application process for these extensions should be broadly accessible. Nominations should not be invitation-only at any career stage, and student awards in particular should allow for self-nomination. A summary of nominations received and the demographics of nominees should be kept and available to society members. The process for appointing selection committees should also be shared, with published guidelines for how the committee is selected and how often members rotate. Selection committees should receive implicit-bias training, and ideally, the composition of the committee will reflect the gender and diversity demographics of society members (Vila-Concejo et al., 2018; Holmes et al., 2020).
2. *Reducing implicit bias in award selection processes.* Double-blind reviews are recommended for student awards. For significant achievement awards, a preliminary anonymous summary of contributions (without reference to the nominee) is advised, although complete anonymity may be challenging in small communities where members are familiar with each other’s work. For nominators, organizations should provide guidance on minimizing bias in recommendation letters and offer examples of gender-neutral letters, as studies have shown men often receive more “superlative” endorsements. Nominations should remain active for multiple award cycles, which reduces the influence of an individual committee or member and allows unsuccessful nominees to be reconsidered by different committees in subsequent cycles (Lincoln et al., 2012).
3. *Strengthen institutional knowledge and communication at smaller conferences.* Smaller, locally organized conferences should implement structures that preserve institutional knowledge and ensure consistent communication between past and current organizers. This could include using conference fees to support a permanent website or similar platform. Improved record-keeping and publication of past award recipients, along with established rubrics for awards and keynote invitations, are also essential. Maintaining records of past recipients helps prevent recognition and speaking opportunities from repeatedly going to the same individuals. Additionally, developing inclusive policies and evaluation rubrics to be shared across conferences reduces the burden on local organizing committees and facilitates consistent, equitable practices. Finally, organizers could maintain records of individuals who decline speaking invitations but express future interest, thereby expanding opportunities for underrepresented groups when circumstances are more conducive.
4. *Expand opportunities for recognition across career stages.* Organizations should establish awards at the student, early-career and mid-career levels, if these do not already exist. Such awards create important opportunities for visibility and can contribute to retention in the discipline (De Welde and Laursen, 2011). Notably, nearly all surveyed organizations lack mid-career awards, resulting in extended gaps between early-career recognition and lifetime or significant achievement awards. The introduction of mid-career awards dedicated to scientific achievement would provide further opportunities for recognition within the growing ranks of female researchers. These awards should clearly define eligibility based on years since the doctorate, have clear policies for extension similar to early-career awards and be explicitly distinguished from lifetime achievement awards.
5. *Annual demographic reporting.* We encourage CGE organizations and conferences to conduct continuous outcome-based monitoring, which includes regular collection and publication of demographic data of the community, keynote speakers, award nominees and recipients (e.g., annually or per conference). Demographic surveys should be fully anonymized and designed to capture both visible and invisible aspects of identity (Clair et al., 2005; Duncombe, 2019). Questions about gender identity, sexual orientation and transgender identity should be asked separately to avoid conflation and allow individuals to self-identify accurately. Such data are essential for ensuring that honors reflect the composition of the relevant CGE sub-community and for evaluating whether inclusive policies are effectively broadening participation and diversifying honorees. These efforts, combined with the recommendations above, support ongoing progress toward gender parity across all career stages.

These data provide a foundation for evaluating gender equity in awards within the CGE community. The community has already made significant progress over the past 25 years compared to the strongly male-dominated first half of our dataset. Continued steps toward transparency in both nomination processes and selection criteria, building institutional knowledge within smaller organizations, creating additional mid-career opportunities and fostering mentoring networks that connect junior women to senior colleagues outside their home institutions – such as the Women in Coastal Geoscience and Engineering organization (e.g., National Academies Press, 2006) – are likely to further advance gender parity in participation and recognition within the CGE community.

## Supporting information

10.1017/cft.2026.10033.sm001 Wilson et al. supplementary material Wilson et al. supplementary material

## Data Availability

The compiled dataset that supports the findings of this study is openly available via Harvard Dataverse at https://doi.org/10.7910/DVN/6MXKMF and the Supplemental Material.

## Supplementary material

The supplementary material for this article can be found at http://doi.org/10.1017/cft.2026.10033.

## Table 1. Long description

From the top row, the American Geophysical Union (A G U) in the United States covers Ocean Sciences and Earth and Planetary Surface Processes, with 28 percent female membership in 2015, awards as recognition, analysis from 1982 to 2024, and 148 entries. Next, the American Shore and Beach Preservation Association (A S B P A) in the United States, awards, 1988 to 2024, 66 entries. The American Society of Civil Engineers (A S C E) in the United States, 17 percent female membership in 2020, awards, 2000 to 2025, 204 entries. Australasian Coasts and Ports (A C and P) in Australia, New Zealand, and Pacific Islands, awards, 2003 to 2023, 42 entries. Australian Coastal Society (A C S) in Australia, Coast to Coast, Queensland Coastal Conference, New South Wales Coastal Conference, 45 percent female membership in 2018, keynotes, 2007 to 2025, 28 entries. Coastal Estuarine Research Foundation (C E R F) in the United States, 20 percent female membership in 2018, awards, 1997 to 2023, 30 entries. Coastal Sediments and Coastal Dynamics Conferences (C S slash C D), international, awards, 2001 to 2025, 15 entries. The Coastal Society in the United States, awards, 2002 to 2024, 40 entries. Coasts, Oceans, Ports and Rivers Institute (C O P R I) in the United States, 15 percent female membership, awards, 1978 to 2025, 111 entries. Community Surface Dynamics Modeling System (C S D M S) in the United States, awards, 2010 to 2025, 27 entries. Estuarine and Coastal Sciences Association (E S C A) in the United Kingdom, 38 percent female membership in 2018, awards, 2012 to 2019, 5 entries. European Geophysical Union (E G U) in Germany and Europe, Ocean Sciences, Geomorphology, Stratigraphy, Sedimentology, Paleontology, 39 percent female membership in 2022, awards, 1991 to 2025, 190 entries. International Conference on Coastal Engineering (I C C E), international, keynotes, 1998 to 2024, 48 entries. National Institute of Maritime, Port, and Aviation Technology Port and Airport Research Institute (N I A) in Japan, awards, 2016 to 2024, 17 entries. New Zealand Coastal Society (N Z C S) in New Zealand, 40 percent female membership in 2018, awards, 2012 to 2024, 31 entries. The World Association for Waterborne Transport Infrastructure (P I A N C), international, awards, 1985 to 2024, 74 entries. Rivers, Coasts and Estuarine Morphodynamics Conference (R C E M), international, keynotes, 1999 to 2025, 78 entries. Female participation statistics are referenced from Vila-Concejo et al. (2018), Lerback and Hanson (2017), American Society of Civil Engineers (2020), and Stadmark et al. (2023).

Navigate back to Table 1..

## Table 2. Long description

Beginning at the top row, the table lists organizations in the first column, followed by award name, style of award, advertisement, nomination categories, evaluation categories, and source. Each cell contains either X for available information, N/A for not applicable, or is left blank for unavailable data. For example, ASBPA awards consistently show X for advertisement, open nomination, nominee eligibility, extension policies, and conflict of interest, but lack details on nominator eligibility and committee composition. ASCE awards provide X for advertisement, open nomination, nominee and nominator eligibility, extension policies, supporting materials, evaluation structure, and conflict of interest, but committee size and composition are often missing. AGU awards include X for most categories, with N/A for nominator role in self-nominated cases. Australasian Coasts and Ports awards display X for advertisement and nominee eligibility, N/A for nominator role, and X for extension policies and conflict of interest. CERF, Coastal Sediments, Coastal Society, COPRI, CSDMS, ECSA, EGU, National Institute of Maritime Port and Aviation Technology, NZCS, and PIANC awards are similarly mapped, with X, N/A, or blanks indicating transparency or lack thereof in each process category. The final column cites the source for each entry. Awards limited to female recipients are marked with an asterisk. Gray cells indicate unavailable information. The table footnote clarifies the meaning of X, N/A, and gray cells, and notes that organizations with five or more awards are grouped together.

Navigate back to Table 2..

## Figure 1. Long description

The top panel shows the distribution of awards by organization, with organizations listed vertically and percentage on the horizontal axis. Each organization has two bars: blue for male and orange for female plus. Most organizations have a higher percentage of male awardees, with CSDMS and NZCS showing the largest female plus representation. The middle panel displays the distribution of awards by career stage, with career stages listed vertically. Late career awards are predominantly male, while early, student, and any career stage awards show a more balanced or female plus distribution. The bottom panel presents the distribution of keynote speakers by organization, with ACS, ICCE, and RCEM listed vertically. All three organizations have a higher percentage of male keynote speakers, with female plus representation below 30 percent in each case. Legends and axis labels are present for clarity.

Navigate back to Figure 1..

## Figure 2. Long description

The x-axis lists years from 1979 to 2023, angled for clarity. The y-axis ranges from 0 to 65, labeled as number of awards given. Each year features a stacked bar: blue for male recipients at the base, orange for female+ above. Early years (1979–1999) show low totals, mostly male. From 2000 onward, both categories increase, with female+ representation growing notably after 2010. Peaks occur around 2017 and 2018, with totals near 65, and a visible decline after 2020. The legend at the top left identifies blue as male and orange as female+.

Navigate back to Figure 2..
