# Awards, keynotes and gender equity in coastal geoscience and engineering: A 50-year perspective – CORRIGENDUM

**DOI:** 10.1017/cft.2026.10040

**Published:** 2026-07-13

**Authors:** Kat Wilson, Sarah Grace Lott, Katherine Anarde

This article was originally published with an incorrect value of 94% in the abstract, impact statement, graphical abstract, and second sentence of the results. This value should be 90%.

The errors in the abstract, impact statement, and graphical abstract have been corrected and the online HTML and PDF versions updated. The error in the second sentence of the results has not been corrected.

The authors apologise for the error.

## References

[r1] Wilson K, Lott SG and Anarde K (2026) Awards, keynotes and gender equity in coastal geoscience and engineering: A 50-year perspective. Cambridge Prisms: Coastal Futures 4, e12. 10.1017/cft.2026.10033 42327847 PMC13276721

