# Supplementary material for: Awards, keynotes and gender equity in coastal geoscience and engineering: A 50-year perspective
Source: Camb Prism Coast Futur. 2026 May 13;4:e12. doi: 10.1017/cft.2026.10033 (PMC13276721; doi:10.1017/cft.2026.10033)
Supplement: Wilson et al. supplementary material [file S275472052610033Xsup001.zip › SM3_SupplementalFigures.pdf]

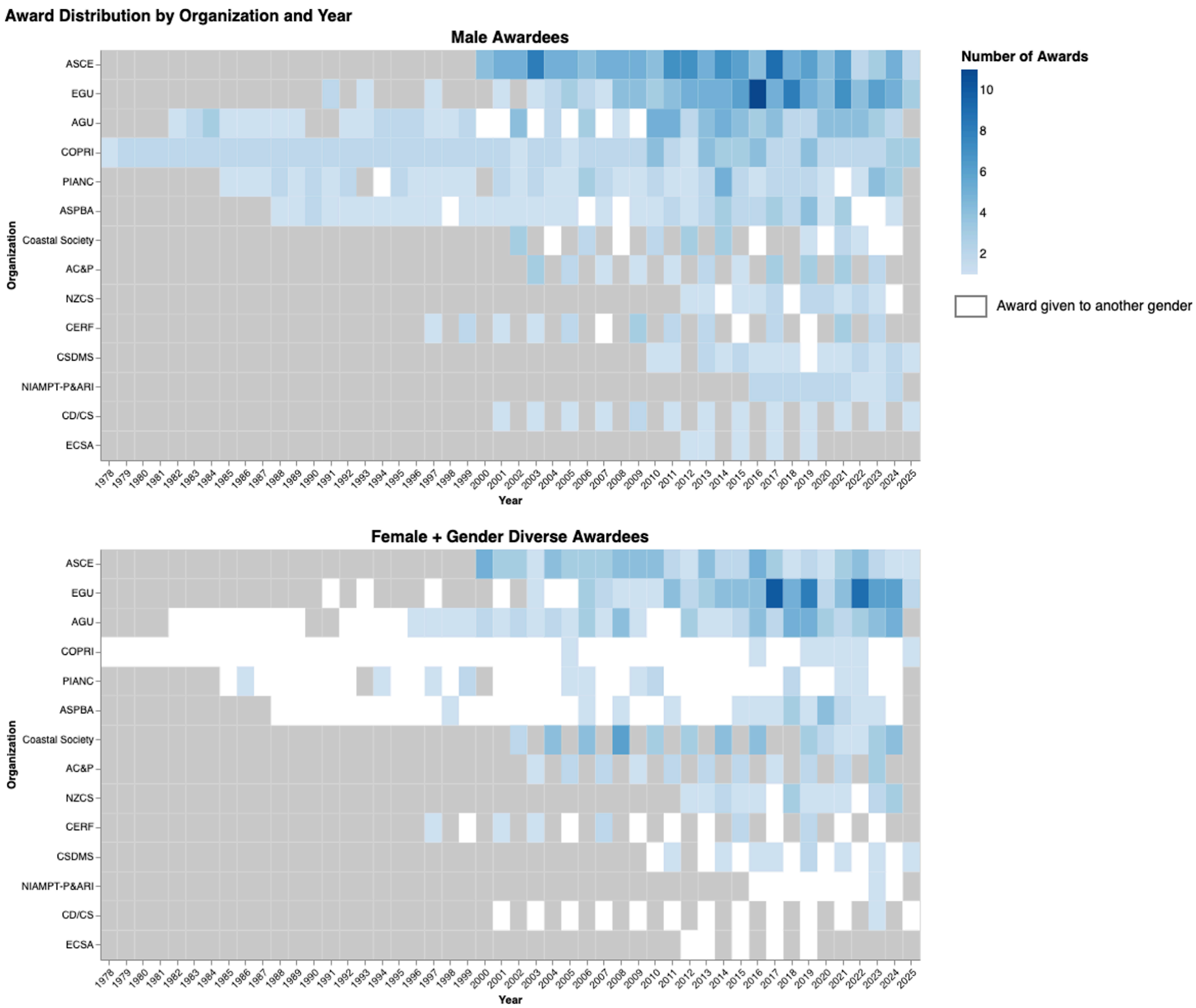

Supplemental Figure 1. Temporal trends in awards given by the surveyed organizations. Gray cells indicate years in which no awards were given. Colors represent the number of awards presented to (A) men or (B) women and gender-diverse awardees. White cells indicate years in which awards were made by the organization but were received by awardees of a different gender category.

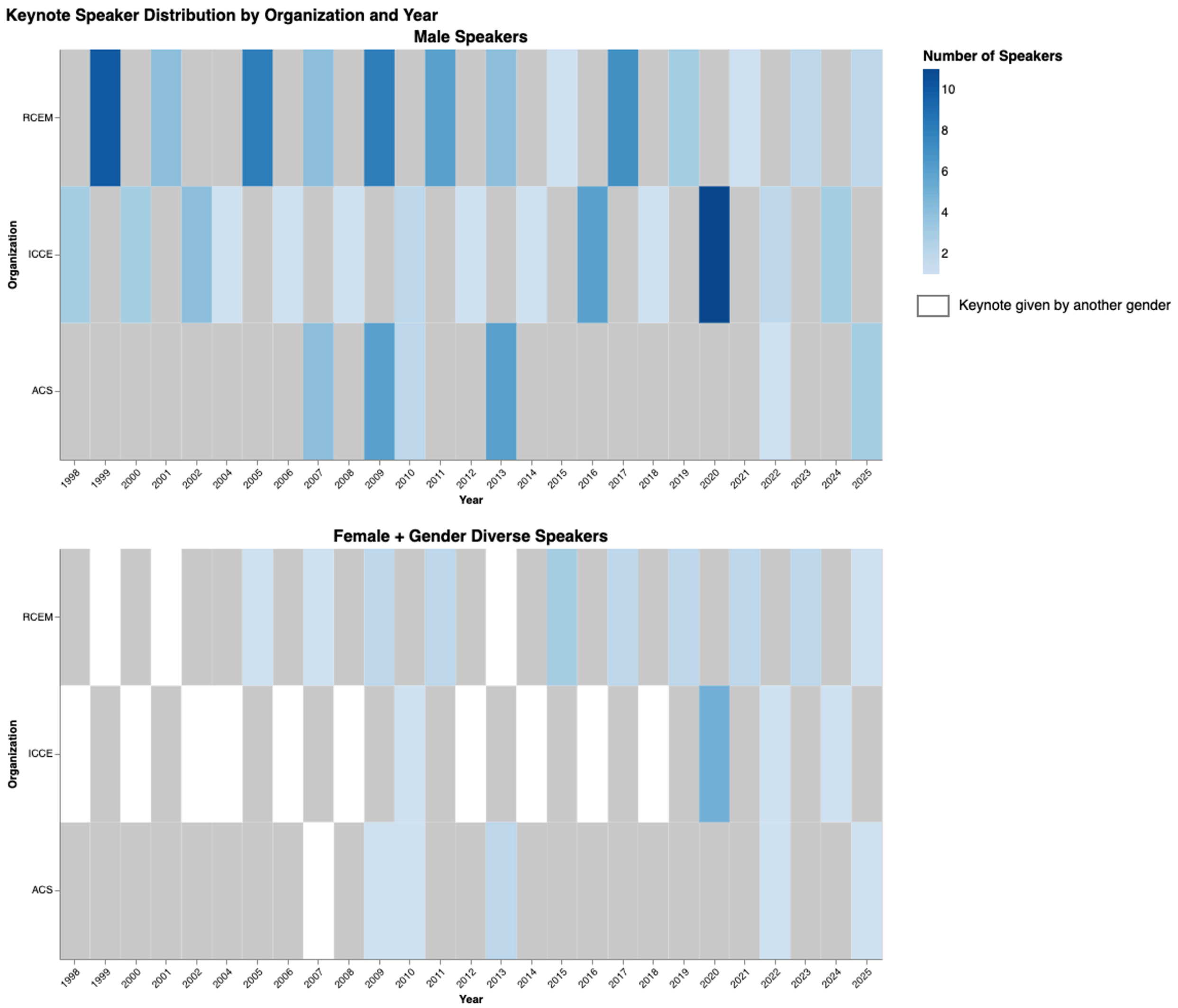

Supplemental Figure 2. Temporal trends in keynotes invited by the surveyed organizations. Gray cells indicate years in which no keynotes were given. Colors represent the number of keynotes presented by (A) men or (B) women and gender-diverse individuals. White cells indicate years in which the conferences and keynotes occurred but were presented by individuals of a different gender category.

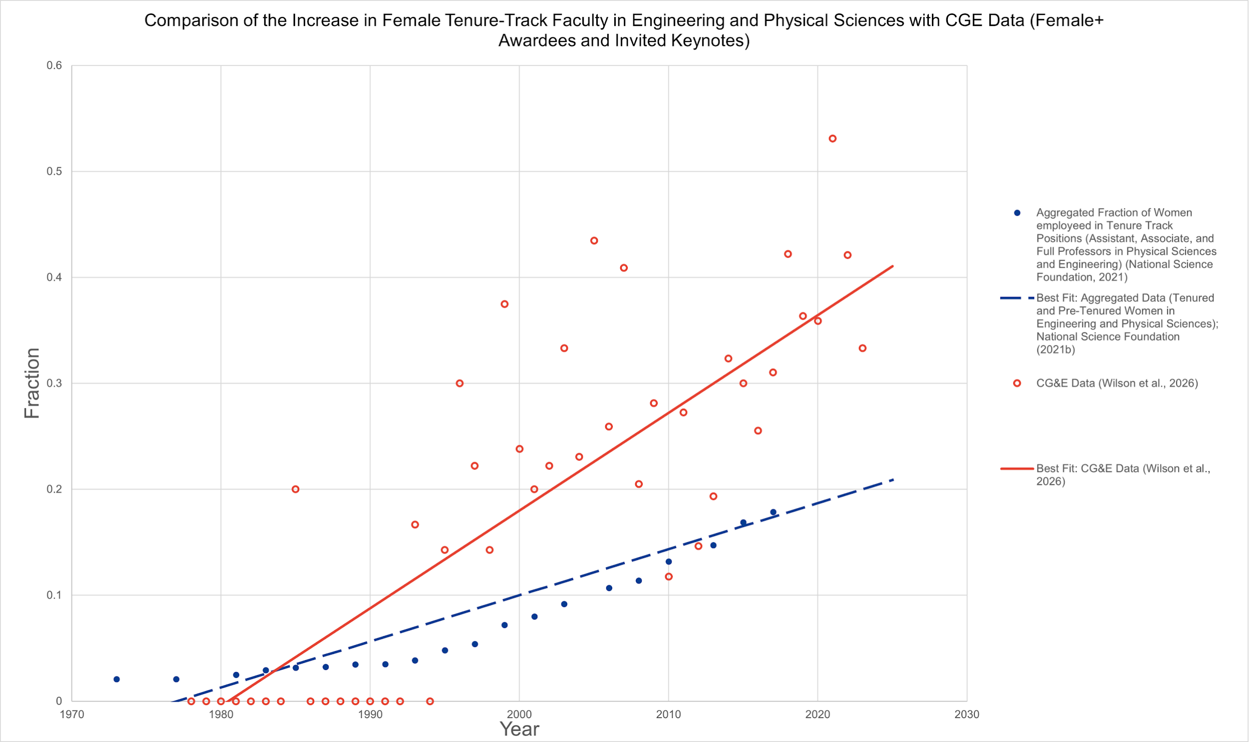

Supplemental Figure 3. Calculated best-fit lines showing the increase in women in tenure-track positions in Engineering and Physical Sciences in the United States (blue dashed line; NSF, 2021) compared to the frequency of female+ awardees and invited keynotes in coastal geoscience and engineering (CGE) compiled for this study (red solid line, Supplemental Material SM1). Student awards were excluded from the comparison.
