# Supplementary material for: Awards, keynotes and gender equity in coastal geoscience and engineering: A 50-year perspective
Source: Camb Prism Coast Futur. 2026 May 13;4:e12. doi: 10.1017/cft.2026.10033 (PMC13276721; doi:10.1017/cft.2026.10033)
Supplement: Peer review history

# Reviewer report: Review: Awards, keynotes and gender equity in coastal geoscience and engineering: A 50-year perspective — R0/PR2

**DOI:** 10.1017/cft.2026.10033.pr2

**Authors:** Suzanne O’Connell

General Assessment

This manuscript presents a rigorous, carefully executed, and valuable synthesis of gender representation in awards and invited keynote recognition across the coastal geoscience and engineering (CGE) community over the past five decades. By assembling and analyzing a large and well-documented dataset (over 1,100 honors), the authors provide the most comprehensive assessment to date of recognition practices in this interdisciplinary field.

The paper is well situated for Cambridge Prisms: Coastal Futures. It bridges quantitative analysis with community reflection, connects historical inequities to present-day outcomes, and offers a constructive, forward-looking discussion that is directly relevant to scientific societies, conference organizers, and senior scholars. Importantly, the authors strike a thoughtful balance between documenting persistent inequities and highlighting genuine progress—particularly the recent acceleration toward gender parity in early- and mid-career recognition.

I support publication of this manuscript. It is methodologically sound, clearly written, and likely to become a useful reference for equity-focused evaluation of recognition practices within CGE and related disciplines.

Major Strengths

1. Scope, Originality, and Data Assembly

The compilation of 50 years of award and keynote data across 14 organizations and multiple conferences is an impressive scholarly contribution in its own right. The authors’ careful documentation of data gaps, classification decisions, and limitations demonstrates methodological transparency and credibility.

2. Clear Framing and Interpretation

The manuscript does an excellent job contextualizing gender representation trends within broader changes in workforce demographics, career-stage distributions, and historical exclusion. The discussion appropriately avoids simplistic causal claims while still offering meaningful interpretation.

3. Balanced Narrative of Progress and Persistence

One of the paper’s most valuable contributions is its nuanced framing: substantial gains since ~2000 are clearly documented, yet persistent disparities—especially in late-career awards and invited keynotes—are not minimized. This balance strengthens the manuscript’s authority and avoids either complacency or overstatement.

4. Community-Relevant Recommendations

The “Paths Forward” section is well grounded in prior literature and in the authors’ empirical findings. The emphasis on transparency, record-keeping, and institutional memory is particularly compelling for smaller conferences and societies.

Primary Consideration: Scope and Practical Burden of Recommendations

The only substantive concern I raise is not with the analysis itself, but with how the recommendations may be perceived by smaller or volunteer-run organizations.

The five recommendations are well justified and thoughtfully articulated. However, taken together, they may appear ambitious—especially for organizations with limited administrative capacity or rotating leadership. This is not a flaw in the science, but a matter of framing. I have sat on many nominating committees and made many nominations and both are time consuming. In most cases in which I was involved, there weren’t that many nominations, which considering the time it takes to build a nomination and the time pressure most people are under is understandable.

With that in mind, I encourage the authors to consider explicitly emphasizing that these recommendations are aspirational and modular, rather than prescriptive or all-or-nothing. For example:

• Clarify that incremental steps (e.g., improved record-keeping alone, or clearer nomination criteria without full demographic reporting) still represent meaningful progress.

• Acknowledge more directly that implementation timelines and feasibility will vary across organizations, particularly those run entirely by volunteers.

• Consider a brief sentence noting that societies may prioritize different recommendations depending on size, resources, and governance structure.

Such framing would preserve the strength of the recommendations while reducing the risk that readers interpret them as unrealistic or overly burdensome.

Importantly, I do not view this as requiring major revision—rather, a small adjustment in tone or emphasis would enhance uptake and goodwill.

Minor Comments and Suggestions

• The figures are clear and effective; Figure 1 in particular provides an intuitive visual summary of disparities by organization and career stage.

• The discussion of keynote selection processes is especially strong and could be highlighted slightly more explicitly as an area of “low-hanging fruit” for improvement.

• The authors’ positionality statement is appropriate and transparently handled; I appreciate its inclusion.

• Probably not for this paper, but I wonder why the Coastal Society has such a strong female representation

Recommendation

Accept with minor revisions.

This is an important, timely, and well-executed contribution that will be of lasting value to the CGE community and beyond. I commend the authors for undertaking such a substantial and careful analysis and strongly encourage publication following minor clarifications regarding the scope and implementation of the recommendations.

# Reviewer report: Review: Awards, keynotes and gender equity in coastal geoscience and engineering: A 50-year perspective — R0/PR3

**DOI:** 10.1017/cft.2026.10033.pr3

It is a very valuable and timely study, which should be published after substantial revision. Revision should help improve reporting transparency and clarity, which are currently suboptimal.

Issues to be addressed:

1. The study has not been registered – this makes it unclear which methods and analyses were specified a priori, and which were added during or after data collection. Were there any prespecified hypotheses for this work?

2. Missing references – the authors should also cite these highly relevant papers:

a. Schroeder J, Dugdale HL, Radersma R, Hinsch M, Buehler DM, Saul J, Porter L, Liker A, De Cauwer I, Johnson PJ, Santure AW, Griffin AS, Bolund E, Ross L, Webb TJ, Feulner PG, Winney I, Szulkin M, Komdeur J, Versteegh MA, Hemelrijk CK, Svensson EI, Edwards H, Karlsson M, West SA, Barrett EL, Richardson DS, van den Brink V, Wimpenny JH, Ellwood SA, Rees M, Matson KD, Charmantier A, Dos Remedios N, Schneider NA, Teplitsky C, Laurance WF, Butlin RK, Horrocks NP. Fewer invited talks by women in evolutionary biology symposia. J Evol Biol. 2013 Sep;26(9):2063-9. doi: 10.1111/jeb.12198. Epub 2013 Jun 20. PMID: 23786459; PMCID: PMC4293461.

b. Jones TM, Fanson KV, Lanfear R, Symonds MR, Higgie M. Gender differences in conference presentations: a consequence of self-selection? PeerJ. 2014 Oct 21;2:e627. doi: 10.7717/peerj.627. PMID: 25346879; PMCID: PMC4207199.

c. Lagisz M, Aich U, Amin B, Rutkowska J, Sánchez-Mercado A, Lara CE, Nakagawa S. Little transparency and equity in scientific awards for early- and mid-career researchers in ecology and evolution. Nat Ecol Evol. 2023 May;7(5):655-665. doi: 10.1038/s41559-023-02028-6. Epub 2023 Apr 3. PMID: 37012379.

d. Lagisz M, Rutkowska J, Aich U, Ross RM, Santana MS, Wang J, Trubanová N, Page MJ, Pua AAY, Yang Y, Amin B, Martinig AR, Barnett A, Surendran A, Zhang J, Borg DN, Elisee J, Wrightson JG, Nakagawa S. “Best Paper” awards lack transparency, inclusivity, and support for Open Science. PLoS Biol. 2024 Jul 23;22(7):e3002715. doi: 10.1371/journal.pbio.3002715. PMID: 39042591; PMCID: PMC11265724.

I note that the questions and methods of this manuscript are strikingly similar to the last two papers in the above list – I wonder of the authors read them before conducting their study and if so how this influenced their study design? I note that the findings and recommendations are also very similar.

3. Methods: “recipients of honors (awards and invited keynotes)” … “we include only scholarly awards “ - Please define “awards” and “invited keynotes”. There are multiple terms used by societies and different specifications of what they consider under each term, what they are intended for and what eligibility rules are. E.g. “awards” are used for nominal recognition (prizes) but also for “grants” (research grants, travel/conference). But there are also “prizes”, “commendations”, “honourable mentions”, “research grants”, “honourable members”, etc. Did you include only specific types of awards, or did you extract any item listed on the website as “award” or similar? Same applies to the “invited keynotes” – they can be also “plenaries”, etc. and sometimes it is usually not clear which ones were invited, and which ones were not and how to determine this? (Ironically, if you apply for a talk at a conference and it gets accepted, you are effectively “invited” to attend and present, so any conference presentation is “invited”…)

4. Methods: “Gender classification of honourees – male or female and gender minorities (herein, “female+”) – was determined using public-facing materials and self-identified pronouns where available.” – please provide more details how you search for this information and whether you used cues other than pronouns and how often.

5. Methods: “Most data came from organizational websites” – please state explicitly what kind of data. Previous paragraph is about gender data so my first impression was that this is also the case here, but reading further down this does not seem so.

6. Methods: “Early-career awards typically specify eligibility within 5 to 10 years of completing a doctoral degree.” – there is often a full or partial overlap with student awards(ie., ECR = student, ECR = student +postdoc) – how often this was the case? Was it coded explicitly?

7. Methods: did you try to break down types of awards – e.g. publications / presentations / overall/contributions/ etc.? My guess is that esp. awards for students and ECR would fall into the first two categories.

8. Results: “Additionally, policies regarding implicit-bias training for selection committees, as recommended by past studies (Lincoln et al., 2011; Holmes et al., 2020), could not be assessed” – explicitly state the reason for this.

9. Results: the service awards have been also compiled – why they are not included in the results?

10. Table 1: “Female attendance and membership statistics” – this is a valuable data collated for this project – it deserves description in the methods and results sections. Somehow, it is used as a baseline in the Impact Statement (but without the context and it appears to be part of the findings there), then in the Intro (as a background), but not in the Abstract or Results, which just feels confusing. Data in Table 2 is mentioned in Methods and Discussion, but not in the Results, where it seems to also fit, as a summary of your data set and some of the findings.

11. Figure 1 “Diverging bar plots of awards in the CGE community” – it looks like the plot shows number of awardees per society not numbers of awards (unless awards have gender). Please be extremely careful how you use word “award” – is this an initiative vs. instances of wining in each round vs. people who got them. Please fix the figure description (labels) too.

12. Make all shared data FAIR (https://www.go-fair.org/fair-principles/). E.g. .csv files instead of xlsx, each data file/table should have detailed meta-data (descriptions of every variable to make the understandable, reusable and replicable). Archive FAIR data in a public repository (e.g. OSF, Zenodo), add licence, get DOI for it and cite the dataset in your manuscript. Some good guides: https://doi.org/10.32942/X24P8S, https://www.openaire.eu/how-to-make-your-data-fair.

# Editor report: Recommendation: Awards, keynotes and gender equity in coastal geoscience and engineering: A 50-year perspective — R0/PR4

**DOI:** 10.1017/cft.2026.10033.pr4

**Affiliations:** School of Civil and Environmental Engineering, UNSW, Australia

Dear Authors,

We have received 2 reviews of your paper that have suggested a range of changes to consider in improving the overall impact and rigour of the work. You will see that R1 (minor) and R2 (major) have both similar and some differing concerns. 

I fully agree with R1’s request to consider the reframing of the end to account for smaller/volunteer organisations. Their suggestion to allow this to be incremental (rather than an all or nothing) is worthwhile. 

R2 provides a much larger critical view of the paper, including requests for more information on methods, etc. They have also included a long list of potential papers to cite, of which I’d like you to consider including at least some that are most relevant to your work. They do raise that the last 2 papers show strong similarities in format/structure to your paper and have asked if you did read them and if these influenced you own design. I think this is a point that must be responded to. If these papers were used as a guide, then I think it appropriate to somewhere in this paper include that in your methods that your study design follows previously published works. I also appreciate that it may also follow similar steps to your own previous paper led by Ana Vila Concejo et al. [Nature Palgrave Communications, 2018]. 

Overall, I have recommended major revisions to provide adequate time to respond to the 2 reviewer comments. I look forward to your revised manuscript and responses. 

Kind regards,

Prof. Kristen Splinter

Senior Editor,

# Editor report: Decision: Awards, keynotes and gender equity in coastal geoscience and engineering: A 50-year perspective — R0/PR5

**DOI:** 10.1017/cft.2026.10033.pr5

**Affiliations:** Geography, Cambridge University, United Kingdom of Great Britain and Northern Ireland

No accompanying comment.

# Reviewer report: Review: Awards, keynotes and gender equity in coastal geoscience and engineering: A 50-year perspective — R1/PR7

**DOI:** 10.1017/cft.2026.10033.pr7

Thank you for revising the manuscript and addressing most of the comments. However, a few issues remain or came out after closer inspection. Specifically:

1. This reply “The suggested breakdown was not done explicitly but was achieved by default because all student awards were for presentations or small scholarships, whereas all non-student awards in the analysis were given for overall contributions.” Did not lead to any changes in the manuscript – please make this breakdown explicit in the manuscript, as currently this is not clear.

2. This reply “We thank the reviewer for this suggestion. The data is now archived in a FAIR-Compliant repository (Harvard Dataverse) with a citation of Wilson et al., 2026 https://doi.org/10.7910/DVN/6MXKMF. Additionally, the data is included as supplementary material as part of the open access publication.” does not satisfy my request for the shared data to be FAIR (the fact that repository is FAIR-compliant is not the same as the archived data being FAIR). I asked for the file to be converted to .csv but it is still an Excel file (with multiple tabs which should be collated in a single table with additional column indicating identity of the society). Also, the data-level metadata is still missing, which means that the dataset is not transparent, reusable or replicable.

3. Table 2: “Table 2: Summary of publicly-available information on nomination and selection processes for organizational awards. Organizations with five or more awards in the database are grouped into a single table entry. …” – please provide (and archive) raw extracted data underlying this table as it is hard to draw conclusions from this one due to the data being aggregated for different types of awards. i.e. create a table where each row corresponds to a single award. Even if “policies were typically shared across awards” some were not shared and will have different characteristics regarding e.g. eligibility or target career stage, especially give that later “Awards are presented by career stage”. Also, please provide links to sources of information of each awards – e.g. websites and whether information was obtained via personal communication with the society. Please provide additional file with metadata – describing how each variable (column) in this table was comment, so that data extraction can be reused and/or reproduced. Share publicly alongside the other data.

4. Line 273: “male-female+” should be “male:female+” (this is how numerical results are formatted in the text, e.g. “(46%:54%)”).

5. Software used for analyses and creating issues is not reported in the manuscript.

6. Code used to calculate numerical values create figures is not publicly shared alongside the data making – i.e. currently this work is not computationally reproducible which should be addressed.

# Editor report: Recommendation: Awards, keynotes and gender equity in coastal geoscience and engineering: A 50-year perspective — R1/PR8

**DOI:** 10.1017/cft.2026.10033.pr8

**Affiliations:** School of Civil and Environmental Engineering, UNSW, Australia

Dear authors, 

We have received 1 review for this paper and I would like to ask if it would be possible to address these so that the underlying data is fully reproducible. My hunch is that others may wish to use your valuable data resource and currently it’s not possible under their definition of FAIR access. Please see the review comments around this. Eg from the reviewer includes turning the xls files (which requires access to MS products) into .csv files.

I note that the reviewer has not made comment related to the specific manuscript itself, but rather the underlying data, codes, and how it is referenced. As such, I feel we should be able to publish this paper very soon. 

I would greatly appreciate if the reviewer suggestions could be addressed to broaden the impact of the paper you have submitted. The reviewer has also asked for codes used to do the analysis, etc. I also understand that the programs that you used/codes may also not be open access and I am not expecting you to transfer analysis to open source programs to satisfy the reviewer. Codes could be put into github. They have asked you to name the programs used. 

I do hope that this is not a large burden on your time to adjust the underlying data for further transparency. 

All the best

Kristen Splinter

Senior Editor

# Editor report: Decision: Awards, keynotes and gender equity in coastal geoscience and engineering: A 50-year perspective — R1/PR9

**DOI:** 10.1017/cft.2026.10033.pr9

**Affiliations:** Geography, Cambridge University, United Kingdom of Great Britain and Northern Ireland

No accompanying comment.

# Editor report: Recommendation: Awards, keynotes and gender equity in coastal geoscience and engineering: A 50-year perspective — R2/PR11

**DOI:** 10.1017/cft.2026.10033.pr11

**Affiliations:** School of Civil and Environmental Engineering, UNSW, Australia

I believe the authors have done what they can regarding addressing the reviewer comments re providing data. I appreciate that the original xls file had background coding in it that the reviewer may not have appreciated. I would suggest that xls has remained as part of the supplementary. I appreciate they have also uploaded a basic csv file as per the R1 request, along with coding where appropriate. I acknowledge and agree to their comment that it would not be feasible to transfer all of their work to something beyond the xls file and coding embedded at this time. 

I recommend the paper is accepted.

# Editor report: Decision: Awards, keynotes and gender equity in coastal geoscience and engineering: A 50-year perspective — R2/PR12

**DOI:** 10.1017/cft.2026.10033.pr12

**Affiliations:** Geography, Cambridge University, United Kingdom of Great Britain and Northern Ireland

No accompanying comment.
